# Rabbit Tularemia and Hepatic Coccidiosis in Wild Rabbit

**DOI:** 10.3201/eid1612.101013

**Published:** 2010-12

**Authors:** Dae Young Kim, Thomas J. Reilly, Susan K. Schommer, Sean T. Spagnoli

**Affiliations:** Author affiliation: University of Missouri, Columbia, Missouri, USA

**Keywords:** Tularemia, hepatic coccidiosis, rabbit, gram-negative bacterium, Francisella tularensis, bacteria, zoonoses, bioterrorism, letter

**To the Editor:** Tularemia is a highly pathogenic zoonosis caused by the gram-negative intracellular bacterium *Francisella tularensis*. *F. tularensis* causes serious septicemia in animals, especially wild rodents and lagomorphs (rabbits and hares), and potentially fatal, multisystemic disease in humans. The human mortality rate can reach 30% in untreated persons (*1*). *F. tularensis* is listed as a category A bioterrorism agent by the Centers for Disease Control and Prevention alongside the causative agents of anthrax, plague, smallpox, botulism, and viral hemorrhagic fevers. Generally, lesions associated with septicemic tularemia include multifocal 1–2-mm, white foci of necrosis in the liver, spleen, lymph nodes, and lungs.

*Eimeria stiedae* is the causative agent of hepatic coccidiosis, a common disease of wild rabbits (*2*) that can result in severe hepatic injury and death in juveniles and neonates. The gross lesion associated with hepatic coccidiosis is unique and nearly pathognomonic. Because *E. stiedae* causes proliferation of bile duct epithelial cells, affected livers contain multifocal, well-demarcated, linear, occasionally branching, bosselated, yellow to pearl-gray lesions that reflect the course of the biliary tree.

We describe a unique case of tularemia in a rabbit co-infected with *E. stiedae*. This case was initially misdiagnosed as simple *E. stiedae* infection on the basis of the classical gross lesions of hepatic coccidiosis, which overshadowed the more subtle tularemia lesions.

A juvenile wild rabbit was brought to a local veterinary clinic for postmortem examination. The owner, located in southwestern Missouri near the Arkansas–Kentucky border, raises wild-captured rabbits in a 10-acre, fenced area reserved for the training of hunting dogs. Beginning in the summer of 2009, a gradual rabbit die-off occurred, progressing to almost complete depopulation by May 2010. The liver from the dead rabbit was submitted to the University of Missouri Veterinary Medical Diagnostic Laboratory (Columbia, MO, USA). Gross examination showed the liver contained multifocal to coalescing, linear, yellow to gray nodules consistent with the classical appearance of hepatic coccidiosis. Although no gross evidence of tularemia was observed, the specimen was treated as potentially infected with tularemia because the veterinarian requested *F. tularensis* testing. Samples were collected and processed for bacteriologic culture, PCR, and histologic evaluation within the confines of a certified biological hood.

The liver contained 2 distinct microscopic lesions. The first was severe biliary hyperplasia with numerous intraepithelial coccidia, consistent with hepatic coccidiosis, as was anticipated. The second, more surprising lesion was an acute, multifocal, necrotizing hepatitis (Figure). The differential diagnoses for acute, multifocal, necrotizing heptatitis in a rabbit include tularemia, Tyzzer disease, listeriosis, and salmonellosis. In this instance, *F. tularensis* was identified by bacterial culture (*3*) and PCR as previously described (*4*). No other pathogenic bacteria were isolated on culture. These results were reported to the veterinarian, the owner, and public health officials. All remaining biological specimens were immediately discarded following the University of Missouri’s select agent protocols, and further analysis was halted, preventing further typing of the isolated *F. tularensis*.

**Figure:**
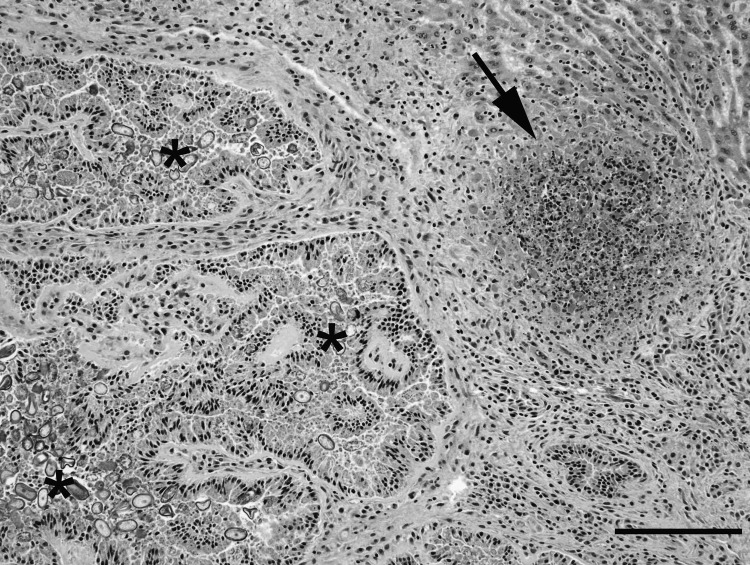
Liver from a juvenile wild rabbit with numerous oval *Eimeria stiedae* oocysts in the convoluted hyperplastic bile ducts (asterisks) and necrotizing hepatitis (arrow) by *Francisella tularensis*. Hematoxylin and eosin stain; scale bar = 200 µm.

According to the Centers for Disease Control and Prevention, ≈126 cases of tularemia are reported annually in the United States (*5*). During 2000–2008, Missouri had the highest number of reported cases (228) followed by Arkansas (149) (*5*). Two subspecies of *F. tularensis* are endemic to the United States: the highly virulent *F. tularensis* subsp. *tularensis* (type A) and the moderately virulent *F. tularensis* subsp. *holarctica* (type B). Transmission of the bacterium occurs primarily through bites from arthropods, including the dog tick (*Dermacentor variabilis*), the wood tick (*D. andersoni*), the lone star tick (*Amblyomma americanum*), and the deer fly (*Chrysops* spp.). In addition, contact with infected animals, most commonly rabbits, wild rodents, and cats, is another common route of transmission to humans (*1**,**6*).

Tularemia occurs in various animal species. Lagomorphs, rodents, and sheep are most susceptible; infected animals are frequently found dead or moribund. Carnivores are less susceptible; however, feline tularemia occurs sporadically, and human infections associated with bites and scratches from infected cats have been recognized (*7*). In addition to arthropod bites, contact with infected dead rabbits or their tissues appears to be the most common source of human infection. A wide variety of case reports have been published describing unique incidences of rabbit–human transmission, including a lawn mower aerosolizing rabbit nests along with their occupants (*8*), consumption of undercooked rabbit meat (*9*), and contact with a “lucky” rabbit’s foot (*10*).

The purpose of this report is to alert veterinarians, veterinary laboratory personnel, and public health officials that rabbit tularemia can be easily overlooked on gross examination in animals displaying lesions of hepatic coccidiosis, a common disease of the wild rabbit. Therefore, all rabbits submitted for postmortem examinations should be regarded as potentially infected with tularemia, particularly during seasons when vectors are active.
